# *trans*-Bis(nitrato-κ*O*)bis­(1,10-phenanthroline-κ^2^ *N*,*N*′)manganese(II)

**DOI:** 10.1107/S1600536812029364

**Published:** 2012-07-04

**Authors:** Watcharin Saphu, Songwuit Chanthee, Kittipong Chainok, David J. Harding, Chaveng Pakawatchai

**Affiliations:** a Department of Chemistry, Faculty of Science, Naresuan University, Muang, Phitsanulok 65000, Thailand; b Molecular Technology Research Unit, Department of Chemistry, Walailak University, Nakhon Si Thammarat 80161, Thailand; c Department of Chemistry, Faculty of Science, Prince of Songkla University, Hat Yai, Songkhla 90112, Thailand

## Abstract

In the title compound, [Mn(NO_3_)_2_(C_12_H_8_N_2_)_2_], the Mn^II^ atom lies on a twofold rotation axis, and is six-coordinated in a distorted *trans*-N_4_O_2_ octa­hedral environment by four N atoms from two 1,10-phenanthroline ligands and two O atoms from two nitrate anions. The nitrate anion is disordered about a twofold rotation axis with fixed occupancy factors of 0.5. In the crystal, mol­ecules are linked by weak C—H⋯O hydrogen bonds and π–π stacking inter­actions [centroid–centroid distance = 4.088 (5) Å] into a three-dimensional supra­molecular network.

## Related literature

For the isotypic Cd compound, see: Shi *et al.* (2004 ▶).
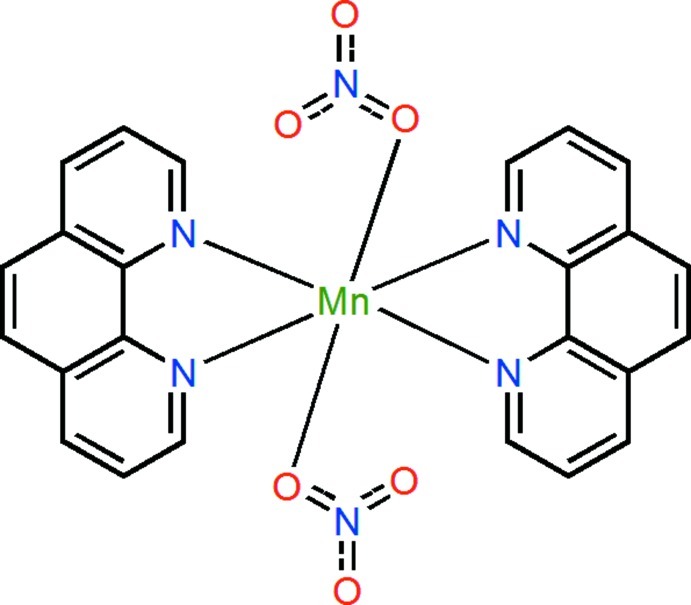


## Experimental

### 

#### Crystal data

[Mn(NO_3_)_2_(C_12_H_8_N_2_)_2_]
*M* *_r_* = 539.37
Monoclinic, 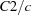

*a* = 11.6191 (6) Å
*b* = 15.1164 (8) Å
*c* = 13.4526 (7) Å
β = 105.387 (1)°
*V* = 2278.1 (2) Å^3^
*Z* = 4
Mo *K*α radiation
μ = 0.64 mm^−1^
*T* = 298 K
0.33 × 0.15 × 0.07 mm

#### Data collection

Bruker SMART CCD area-detector diffractometer
Absorption correction: multi-scan (*SADABS*; Sheldrick, 1996 ▶) *T*_min_ = 0.819, *T*_max_ = 1.000
13250 measured reflections
2748 independent reflections
2367 reflections with *I* > 2σ(*I*)
*R*_int_ = 0.028

#### Refinement

*R*[*F* ^2^ > 2σ(*F* ^2^)] = 0.045
*wR*(*F* ^2^) = 0.101
*S* = 1.15
2748 reflections
204 parameters
60 restraints
H-atom parameters constrained
Δρ_max_ = 0.32 e Å^−3^
Δρ_min_ = −0.19 e Å^−3^

### 

Data collection: *SMART* (Bruker, 1998 ▶); cell refinement: *SAINT* (Bruker, 2003 ▶); data reduction: *SAINT*; program(s) used to solve structure: *SHELXS97* (Sheldrick, 2008 ▶); program(s) used to refine structure: *SHELXL97* (Sheldrick, 2008 ▶); molecular graphics: *DIAMOND* (Brandenburg, 2006 ▶); software used to prepare material for publication: *publCIF* (Westrip, 2010 ▶).

## Supplementary Material

Crystal structure: contains datablock(s) global, I. DOI: 10.1107/S1600536812029364/ng5277sup1.cif

Structure factors: contains datablock(s) I. DOI: 10.1107/S1600536812029364/ng5277Isup2.hkl

Supplementary material file. DOI: 10.1107/S1600536812029364/ng5277Isup3.cdx

Additional supplementary materials:  crystallographic information; 3D view; checkCIF report

## Figures and Tables

**Table 1:** Hydrogen-bond geometry (Å, °)

*D*—H⋯*A*	*D*—H	H⋯*A*	*D*⋯*A*	*D*—H⋯*A*
C3—H3⋯O1*A* ^i^	0.93	2.40	3.232 (9)	148
C3—H3⋯O1*B* ^i^	0.93	2.40	3.214 (10)	146
C7—H7⋯O2*A* ^ii^	0.93	2.29	3.101 (8)	146

Symmetry codes: (i) 
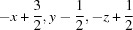
; (ii) 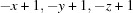.

**Fig. 1.**
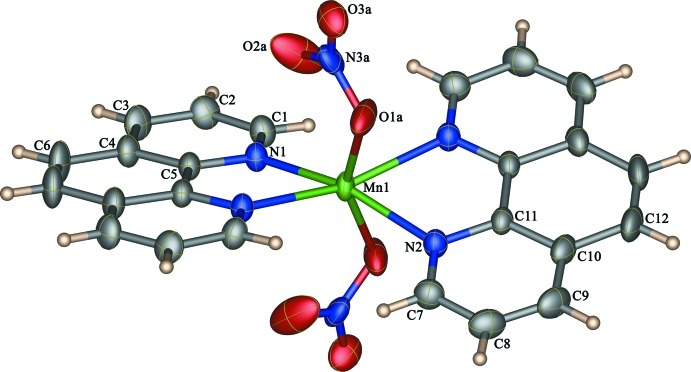
Displacement ellipsoid plot of a fragment at the 30% probability level containing the asymmetric unit with atom numbering and coordination environment of the metal centers of I.

**Fig. 2.**
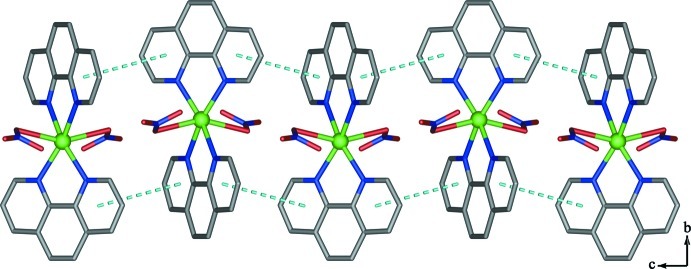
The one dimensional supramolecular chain linked by intermolecular π–π stacking interactions in I.

**Fig. 3.**
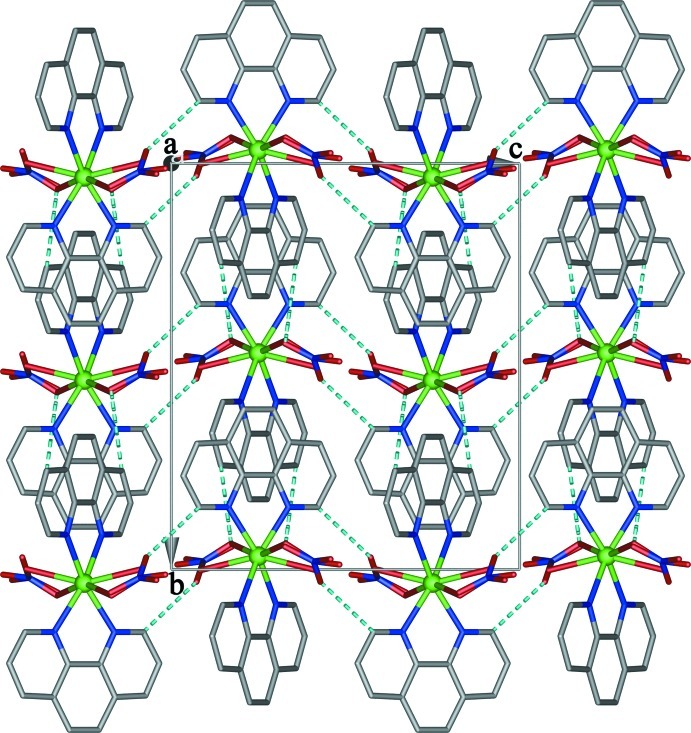
The stacking plot of I, showing H-bond interactions (dashed lines) and π–π stacking interactions along the a axis.

## supplementary crystallographic information

### Comment

The mononuclear metal complexes of the chelating bidentate 1,10-phenanthroline (phen) and 2,2'-bipyridine (bipy) ligands are well known in the literature, and have been used in many fields. In the realm of coordination polymers, these complexes have been employed as coordination acceptor nodes for the construction of low dimensional polymer-based magnets exhibiting long-range magnetic ordering and spin crossover transitions. This communication forms part of our study of the synthesis and magnetic properties of one dimensional tube-like cyanide-bridged bimetallic coordination polymers. The main strategy of the proposed tube motif is to combine two building blocks involving one coordination donor, the chelated tetracyanoferrate, [Fe(*L*)(CN)_4_]*^x^*^-^ (*x* = 1 or 2), and a coordination acceptor, [*M*(*L*)S] (where *M* = an octahedral metal; *L* = bipy or phen, S = solvents or counter ions). Here, we describe the crystal structure of a building block *trans*-[Mn(phen)_2_(NO_3_)_2_] (I), which is a new member of the mononuclear metal complexes with chelating bidentate ligands.

Compound I is isostructural with the Cd analog (Shi *et al.*, 2004). It crystallizes in a monoclinic system in the space group *C*2/*c*, and contains half of the complex molecule per asymmetric unit, Fig. 1. The Mn^II^ atom lies on a twofold rotation axis, and is six-coordinate in a distorted *trans*-MnN_4_O_2_ octahedral environment by two O atoms from two disordered [NO_3_]^–^ anions and four N atoms from two phen ligands. The dihedral angle between the least-squares planes of the two phen ligands [maximum deviation = 0.036 (1) Å] is 25.01 (5)°.

In the crystal, molecules are assembled into one dimensional supramolecular chains parallel to the *c* axis through weak π–π stacking between adjacent aromatic rings of the phen ligands with a centroid-centroid distance of 4.088 (5) Å, Fig. 2. Weak C—H···O hydrogen bonds involving the phen ligands and the [NO_3_]^–^ anions, Table 1, are further linked to neighboring chains into a three dimensional supramolecular network along the *a* axis, Fig. 3.

### Experimental

A methanol solution (5 ml) of phen (20.0 mg, 0.1 mmol) was added dropwise to a methanol solution (5 ml) of Mn(NO_3_)_2_.4H_2_O (30.0 mg, 0.1 mmol) with constant stirring 1 h and filtered to remove any undissolved solid. The filtrate was allowed to stand to slowly evaporate, at room temperature. After one week, yellow blocks of I were obtained (Yield 23 mg, 88% based on Mn source). IR *υ*_max_(cm^-1^): 722 s, 768w, 780w, 821w, 842*m*, 853w, 863w, 1017*m*, 1048w, 1101w, 1141br, 1223w, 1290*m*, 1325*m*, 1408w, 1427w, 1459w, 1518*m*, 1578w, 1624w, 3059br. UV-Vis (CH_3_OH/H_2_O), λ_max_ = 265 nm.

### Refinement

The C-bound hydrogen atoms were placed in geometrically idealized positions based on chemical coordinations and constrained to ride on their parent atom positions with a C–H distances of 0.93 Å and with *U*_iso_(H) = 1.2*U*_eq_(C) for the aromatic H atoms. The nitrate anions are disordered about a twofold rotation axis and were refined using a two site model. The site occupancy factors for the two orientations were then fixed to 0.5. The nitrogen-oxygen distances were restrained to 1.24 ± 0.01 Å and O···O of 2.15 Å, however, the anisotropic temperature factors were restrained to be nearly isotropic.

### Crystal data

**Table:**

[Mn(NO_3_)_2_(C_12_H_8_N_2_)_2_]	*F*(000) = 1100
*M**_r_* = 539.37	*D*_x_ = 1.573 Mg m^−^^3^
Monoclinic, *C*2/*c*	Mo *K*α radiation, λ = 0.71073 Å
*a* = 11.6191 (6) Å	Cell parameters from 3448 reflections
*b* = 15.1164 (8) Å	θ = 2.5–23.1°
*c* = 13.4526 (7) Å	µ = 0.64 mm^−^^1^
β = 105.387 (1)°	*T* = 298 K
*V* = 2278.1 (2) Å^3^	Block, yellow
*Z* = 4	0.33 × 0.15 × 0.07 mm

### Data collection

**Table:**

Bruker SMART CCD area-detector diffractometer	2748 independent reflections
Radiation source: fine-focus sealed tube	2367 reflections with *I* > 2σ(*I*)
Graphite monochromator	*R*_int_ = 0.028
Detector resolution: 8 pixels mm^-1^	θ_max_ = 28.1°, θ_min_ = 2.3°
ω and φ scans	*h* = −15→15
Absorption correction: multi-scan (*SADABS*; Sheldrick, 1996)	*k* = −20→20
*T*_min_ = 1.000, *T*_max_ = 0.819	*l* = −17→17
13250 measured reflections	

### Refinement

**Table:**

Refinement on *F*^2^	Primary atom site location: structure-invariant direct methods
Least-squares matrix: full	Secondary atom site location: difference Fourier map
*R*[*F*^2^ > 2σ(*F*^2^)] = 0.045	Hydrogen site location: inferred from neighbouring sites
*wR*(*F*^2^) = 0.101	H-atom parameters constrained
*S* = 1.15	*w* = 1/[σ^2^(*F*_o_^2^) + (0.0483*P*)^2^ + 0.5759*P*] where *P* = (*F*_o_^2^ + 2*F*_c_^2^)/3
2748 reflections	(Δ/σ)_max_ < 0.001
204 parameters	Δρ_max_ = 0.32 e Å^−^^3^
60 restraints	Δρ_min_ = −0.19 e Å^−^^3^

### Special details

**Table:**

Geometry. All e.s.d.'s (except the e.s.d. in the dihedral angle between two l.s. planes) are estimated using the full covariance matrix. The cell e.s.d.'s are taken into account individually in the estimation of e.s.d.'s in distances, angles and torsion angles; correlations between e.s.d.'s in cell parameters are only used when they are defined by crystal symmetry. An approximate (isotropic) treatment of cell e.s.d.'s is used for estimating e.s.d.'s involving l.s. planes.
Refinement. Refinement of *F*^2^ against all reflections. The weighted *R*-factor *wR* and goodness of fit *S* are based on *F*^2^, conventional *R*-factors *R* are based on *F*, with *F* set to zero for negative *F*^2^. The threshold expression of *F*^2^ > σ(*F*^2^) is used only for calculating *R*-factors(gt) *etc*. and is not relevant to the choice of reflections for refinement. *R*-factors based on *F*^2^ are statistically about twice as large as those based on *F*, and *R*- factors based on ALL data will be even larger.

### Fractional atomic coordinates and isotropic or equivalent isotropic displacement parameters (Å^2^)

**Table:**

	*x*	*y*	*z*	*U*_iso_*/*U*_eq_	Occ. (<1)
Mn1	0.5000	0.53331 (2)	0.2500	0.04311 (14)	
O2B	0.6274 (5)	0.4964 (4)	0.4166 (6)	0.0606 (12)	0.50
N3B	0.7282 (5)	0.5186 (4)	0.4076 (6)	0.039 (2)	0.50
N1	0.58698 (13)	0.41378 (9)	0.19902 (11)	0.0433 (3)	
C1	0.67011 (17)	0.41303 (13)	0.14807 (15)	0.0509 (4)	
H1	0.6993	0.4670	0.1322	0.061*	
N2	0.46192 (14)	0.65384 (10)	0.33693 (12)	0.0470 (4)	
C2	0.71604 (18)	0.33619 (14)	0.11701 (17)	0.0575 (5)	
H2	0.7726	0.3390	0.0795	0.069*	
C3	0.67701 (19)	0.25688 (14)	0.14224 (18)	0.0608 (5)	
H3	0.7077	0.2047	0.1231	0.073*	
C4	0.59018 (18)	0.25400 (12)	0.19736 (16)	0.0533 (5)	
C5	0.54589 (15)	0.33464 (11)	0.22300 (14)	0.0423 (4)	
C6	0.5430 (2)	0.17310 (14)	0.2252 (2)	0.0742 (7)	
H6	0.5724	0.1195	0.2085	0.089*	
C7	0.42093 (19)	0.65392 (15)	0.41958 (16)	0.0584 (5)	
H7	0.4096	0.5998	0.4485	0.070*	
C8	0.3938 (2)	0.73067 (18)	0.46553 (18)	0.0684 (6)	
H8	0.3632	0.7275	0.5227	0.082*	
C9	0.4125 (2)	0.81020 (16)	0.42592 (17)	0.0702 (7)	
H9	0.3957	0.8622	0.4564	0.084*	
C10	0.45699 (18)	0.81377 (13)	0.33906 (16)	0.0571 (5)	
C11	0.47871 (16)	0.73301 (11)	0.29567 (14)	0.0438 (4)	
C12	0.4796 (2)	0.89451 (13)	0.29208 (19)	0.0760 (7)	
H12	0.4658	0.9482	0.3207	0.091*	
O1A	0.6994 (7)	0.5637 (6)	0.3267 (6)	0.087 (3)	0.50
N3A	0.7310 (8)	0.5230 (6)	0.4089 (7)	0.077 (4)	0.50
O2A	0.6590 (8)	0.4707 (6)	0.4282 (7)	0.136 (4)	0.50
O3A	0.8353 (6)	0.5253 (5)	0.4576 (7)	0.0747 (19)	0.50
O1B	0.7286 (8)	0.5562 (6)	0.3270 (6)	0.096 (3)	0.50
O3B	0.8112 (7)	0.5128 (8)	0.4831 (6)	0.105 (4)	0.50

### Atomic displacement parameters (Å^2^)

**Table:**

	*U*^11^	*U*^22^	*U*^33^	*U*^12^	*U*^13^	*U*^23^
Mn1	0.0543 (2)	0.02415 (19)	0.0546 (3)	0.000	0.02096 (18)	0.000
O2B	0.0571 (19)	0.063 (2)	0.065 (3)	0.0025 (19)	0.0224 (17)	0.0032 (19)
N3B	0.038 (4)	0.020 (3)	0.060 (6)	0.008 (2)	0.017 (3)	−0.003 (3)
N1	0.0468 (8)	0.0325 (7)	0.0534 (8)	0.0005 (6)	0.0183 (6)	0.0011 (6)
C1	0.0518 (10)	0.0441 (10)	0.0614 (11)	0.0033 (8)	0.0229 (9)	0.0061 (8)
N2	0.0570 (9)	0.0363 (8)	0.0506 (9)	0.0010 (6)	0.0194 (7)	−0.0003 (6)
C2	0.0534 (11)	0.0563 (12)	0.0691 (13)	0.0114 (9)	0.0276 (10)	0.0016 (10)
C3	0.0657 (12)	0.0451 (11)	0.0764 (14)	0.0169 (9)	0.0273 (11)	−0.0039 (10)
C4	0.0608 (11)	0.0340 (9)	0.0666 (12)	0.0068 (8)	0.0193 (10)	0.0004 (8)
C5	0.0471 (9)	0.0308 (8)	0.0489 (10)	0.0011 (7)	0.0128 (8)	0.0004 (7)
C6	0.0909 (18)	0.0293 (10)	0.112 (2)	0.0061 (10)	0.0432 (15)	−0.0026 (11)
C7	0.0636 (12)	0.0593 (13)	0.0563 (12)	−0.0013 (9)	0.0229 (10)	0.0002 (9)
C8	0.0662 (13)	0.0844 (18)	0.0570 (12)	0.0140 (12)	0.0206 (10)	−0.0108 (12)
C9	0.0742 (14)	0.0644 (15)	0.0671 (14)	0.0244 (11)	0.0100 (11)	−0.0214 (11)
C10	0.0616 (12)	0.0383 (10)	0.0639 (12)	0.0112 (8)	0.0032 (10)	−0.0098 (9)
C11	0.0459 (9)	0.0308 (8)	0.0515 (10)	0.0020 (7)	0.0075 (7)	−0.0021 (7)
C12	0.0973 (18)	0.0293 (10)	0.0893 (17)	0.0069 (10)	0.0033 (14)	−0.0102 (9)
O1A	0.091 (4)	0.043 (3)	0.097 (6)	−0.012 (2)	−0.027 (3)	0.015 (3)
N3A	0.099 (9)	0.068 (6)	0.069 (8)	−0.030 (5)	0.031 (7)	−0.036 (5)
O2A	0.191 (8)	0.154 (8)	0.083 (5)	−0.117 (6)	0.070 (6)	−0.023 (5)
O3A	0.062 (3)	0.065 (3)	0.088 (4)	0.013 (2)	0.003 (3)	−0.016 (3)
O1B	0.154 (7)	0.075 (5)	0.085 (5)	−0.060 (4)	0.076 (5)	−0.021 (3)
O3B	0.062 (3)	0.162 (8)	0.085 (5)	0.027 (4)	0.004 (3)	−0.050 (5)

### Geometric parameters (Å, º)

**Table:**

Mn1—N1	2.2636 (14)	C3—H3	0.9300
Mn1—N1^i^	2.2636 (14)	C4—C5	1.401 (2)
Mn1—N2	2.2711 (15)	C4—C6	1.430 (3)
Mn1—N2^i^	2.2712 (15)	C5—C5^i^	1.441 (3)
Mn1—O1A	2.318 (8)	C6—C6^i^	1.340 (5)
Mn1—O1A^i^	2.318 (8)	C6—H6	0.9300
Mn1—O2B^i^	2.401 (7)	C7—C8	1.389 (3)
Mn1—O2B	2.401 (7)	C7—H7	0.9300
O2B—N3B	1.255 (6)	C8—C9	1.356 (4)
N3B—O3B	1.204 (7)	C8—H8	0.9300
N3B—O1B	1.225 (6)	C9—C10	1.399 (3)
N1—C1	1.324 (2)	C9—H9	0.9300
N1—C5	1.358 (2)	C10—C11	1.405 (2)
C1—C2	1.388 (3)	C10—C12	1.431 (3)
C1—H1	0.9300	C11—C11^i^	1.441 (4)
N2—C7	1.321 (2)	C12—C12^i^	1.338 (5)
N2—C11	1.355 (2)	C12—H12	0.9300
C2—C3	1.357 (3)	O1A—N3A	1.233 (7)
C2—H2	0.9300	N3A—O3A	1.216 (7)
C3—C4	1.402 (3)	N3A—O2A	1.227 (7)
			
N1—Mn1—N1^i^	74.08 (7)	C7—N2—Mn1	126.68 (14)
N1—Mn1—N2	163.74 (5)	C11—N2—Mn1	115.38 (12)
N1^i^—Mn1—N2	108.71 (5)	C3—C2—C1	118.91 (19)
N1—Mn1—N2^i^	108.71 (5)	C3—C2—H2	120.5
N1^i^—Mn1—N2^i^	163.74 (5)	C1—C2—H2	120.5
N2—Mn1—N2^i^	73.31 (8)	C2—C3—C4	119.66 (18)
N1—Mn1—O1A	79.6 (3)	C2—C3—H3	120.2
N1^i^—Mn1—O1A	119.80 (18)	C4—C3—H3	120.2
N2—Mn1—O1A	85.4 (3)	C5—C4—C3	117.76 (17)
N2^i^—Mn1—O1A	76.24 (18)	C5—C4—C6	119.20 (19)
N1—Mn1—O1A^i^	119.80 (18)	C3—C4—C6	123.00 (18)
N1^i^—Mn1—O1A^i^	79.6 (3)	N1—C5—C4	122.18 (16)
N2—Mn1—O1A^i^	76.24 (18)	N1—C5—C5^i^	118.26 (9)
N2^i^—Mn1—O1A^i^	85.4 (3)	C4—C5—C5^i^	119.56 (11)
O1A—Mn1—O1A^i^	157.2 (4)	C6^i^—C6—C4	121.23 (12)
N1—Mn1—O2B^i^	75.28 (17)	C6^i^—C6—H6	119.4
N1^i^—Mn1—O2B^i^	83.30 (16)	C4—C6—H6	119.4
N2—Mn1—O2B^i^	120.73 (17)	N2—C7—C8	123.4 (2)
N2^i^—Mn1—O2B^i^	82.05 (17)	N2—C7—H7	118.3
O1A—Mn1—O2B^i^	139.2 (3)	C8—C7—H7	118.3
O1A^i^—Mn1—O2B^i^	48.3 (3)	C9—C8—C7	119.1 (2)
N1—Mn1—O2B	83.30 (16)	C9—C8—H8	120.4
N1^i^—Mn1—O2B	75.28 (17)	C7—C8—H8	120.4
N2—Mn1—O2B	82.04 (17)	C8—C9—C10	119.7 (2)
N2^i^—Mn1—O2B	120.73 (17)	C8—C9—H9	120.1
O1A—Mn1—O2B	48.3 (3)	C10—C9—H9	120.1
O1A^i^—Mn1—O2B	139.2 (3)	C9—C10—C11	117.4 (2)
O2B^i^—Mn1—O2B	153.1 (3)	C9—C10—C12	123.7 (2)
N3B—O2B—Mn1	102.0 (5)	C11—C10—C12	118.9 (2)
O3B—N3B—O1B	126.1 (7)	N2—C11—C10	122.41 (18)
O3B—N3B—O2B	117.3 (7)	N2—C11—C11^i^	117.94 (10)
O1B—N3B—O2B	115.6 (6)	C10—C11—C11^i^	119.64 (12)
C1—N1—C5	117.77 (15)	C12^i^—C12—C10	121.45 (13)
C1—N1—Mn1	127.53 (12)	C12^i^—C12—H12	119.3
C5—N1—Mn1	114.70 (11)	C10—C12—H12	119.3
N1—C1—C2	123.67 (18)	N3A—O1A—Mn1	108.9 (7)
N1—C1—H1	118.2	O3A—N3A—O2A	122.9 (9)
C2—C1—H1	118.2	O3A—N3A—O1A	119.0 (9)
C7—N2—C11	117.87 (17)	O2A—N3A—O1A	117.0 (9)
			
N1—Mn1—O2B—N3B	72.3 (5)	N1—C1—C2—C3	−2.0 (3)
N1^i^—Mn1—O2B—N3B	147.6 (5)	C1—C2—C3—C4	1.0 (3)
N2—Mn1—O2B—N3B	−100.6 (5)	C2—C3—C4—C5	0.9 (3)
N2^i^—Mn1—O2B—N3B	−35.5 (5)	C2—C3—C4—C6	178.8 (2)
O1A—Mn1—O2B—N3B	−9.7 (5)	C1—N1—C5—C4	1.2 (3)
O1A^i^—Mn1—O2B—N3B	−158.6 (5)	Mn1—N1—C5—C4	−179.84 (14)
O2B^i^—Mn1—O2B—N3B	109.4 (5)	C1—N1—C5—C5^i^	−179.05 (19)
Mn1—O2B—N3B—O3B	175.4 (8)	Mn1—N1—C5—C5^i^	−0.1 (3)
Mn1—O2B—N3B—O1B	5.9 (9)	C3—C4—C5—N1	−2.1 (3)
N1^i^—Mn1—N1—C1	178.83 (19)	C6—C4—C5—N1	179.9 (2)
N2—Mn1—N1—C1	−78.8 (3)	C3—C4—C5—C5^i^	178.2 (2)
N2^i^—Mn1—N1—C1	15.61 (17)	C6—C4—C5—C5^i^	0.2 (3)
O1A—Mn1—N1—C1	−55.9 (2)	C5—C4—C6—C6^i^	0.3 (5)
O1A^i^—Mn1—N1—C1	111.2 (3)	C3—C4—C6—C6^i^	−177.7 (3)
O2B^i^—Mn1—N1—C1	91.7 (2)	C11—N2—C7—C8	0.4 (3)
O2B—Mn1—N1—C1	−104.6 (2)	Mn1—N2—C7—C8	−176.49 (16)
N1^i^—Mn1—N1—C5	0.05 (9)	N2—C7—C8—C9	−1.6 (4)
N2—Mn1—N1—C5	102.4 (2)	C7—C8—C9—C10	0.8 (3)
N2^i^—Mn1—N1—C5	−163.17 (12)	C8—C9—C10—C11	1.0 (3)
O1A—Mn1—N1—C5	125.4 (2)	C8—C9—C10—C12	−179.9 (2)
O1A^i^—Mn1—N1—C5	−67.6 (3)	C7—N2—C11—C10	1.6 (3)
O2B^i^—Mn1—N1—C5	−87.1 (2)	Mn1—N2—C11—C10	178.82 (14)
O2B—Mn1—N1—C5	76.6 (2)	C7—N2—C11—C11^i^	−178.8 (2)
C5—N1—C1—C2	0.8 (3)	Mn1—N2—C11—C11^i^	−1.6 (3)
Mn1—N1—C1—C2	−177.93 (15)	C9—C10—C11—N2	−2.3 (3)
N1—Mn1—N2—C7	−82.9 (3)	C12—C10—C11—N2	178.55 (19)
N1^i^—Mn1—N2—C7	14.44 (18)	C9—C10—C11—C11^i^	178.2 (2)
N2^i^—Mn1—N2—C7	177.5 (2)	C12—C10—C11—C11^i^	−1.0 (3)
O1A—Mn1—N2—C7	−105.5 (2)	C9—C10—C12—C12^i^	−178.8 (3)
O1A^i^—Mn1—N2—C7	88.2 (3)	C11—C10—C12—C12^i^	0.3 (4)
O2B^i^—Mn1—N2—C7	107.8 (2)	N1—Mn1—O1A—N3A	−82.7 (8)
O2B—Mn1—N2—C7	−57.0 (2)	N1^i^—Mn1—O1A—N3A	−17.9 (9)
N1—Mn1—N2—C11	100.2 (2)	N2—Mn1—O1A—N3A	91.0 (8)
N1^i^—Mn1—N2—C11	−162.48 (12)	N2^i^—Mn1—O1A—N3A	164.9 (8)
N2^i^—Mn1—N2—C11	0.58 (9)	O1A^i^—Mn1—O1A—N3A	127.4 (8)
O1A—Mn1—N2—C11	77.6 (2)	O2B^i^—Mn1—O1A—N3A	−135.2 (7)
O1A^i^—Mn1—N2—C11	−88.7 (3)	O2B—Mn1—O1A—N3A	7.6 (7)
O2B^i^—Mn1—N2—C11	−69.2 (2)	Mn1—O1A—N3A—O3A	175.0 (8)
O2B—Mn1—N2—C11	126.1 (2)	Mn1—O1A—N3A—O2A	6.7 (13)

Symmetry code: (i) −*x*+1, *y*, −*z*+1/2.

### Hydrogen-bond geometry (Å, º)

**Table:**

*D*—H···*A*	*D*—H	H···*A*	*D*···*A*	*D*—H···*A*
C3—H3···O1*A*^ii^	0.93	2.40	3.232 (9)	148
C3—H3···O1*B*^ii^	0.93	2.40	3.214 (10)	146
C7—H7···O2*A*^iii^	0.93	2.29	3.101 (8)	146

Symmetry codes: (ii) −*x*+3/2, *y*−1/2, −*z*+1/2; (iii) −*x*+1, −*y*+1, −*z*+1.
